# The impact of entertainment screen time on sleep quality in Chinese and British adolescents: a moderated mediation model

**DOI:** 10.3389/fpsyg.2025.1612686

**Published:** 2026-01-05

**Authors:** Jingjing Yang, Shanshan Zhang

**Affiliations:** 1 School of Vocational Education, Tianjin University of Technology and Education, Tianjin, China; 2 Key Research Institute of Humanities and Social Sciences at Tianjin Universities—Center of Vocational Education Development Research, Tianjin, China

**Keywords:** screen time, self-control, sleep quality, adolescents, culture

## Abstract

**Introduction:**

Adolescents spend substantial time on media devices for entertainment. Sleep-a critical factor for physical and mental development-is increasingly insufficient among them. One of the contributing factors is the increased availability of technology. Despite previous studies indicating a link between screen time and sleep impairment, the psychological mechanisms underlying this relationship remain unclear. Additionally, the role of culture in this context is still ambiguous. This study aimed to investigate a conceptual framework elucidating how screen time influences sleep quality in adolescents through the mediation role of self-control, with country culture as a moderator.

**Methods:**

A total of 731 Chinese and British adolescents (mean age = 13.01 years, SD = 0.98) completed screen time scale, Children's Perceived Self-control Scale, and part of Youth Self-Rating Insomnia Scale (YSIS) at first. After the removal of values didn't meet criteria, 640 students (mean age = 12.93 years, SD =0.96) were retained for data analysis.

**Results:**

The results showed that (a) screen time was negatively associated with sleep quality (b = 0.22, p < 0.001); (b) self-control mediated the relationship between screen time and sleep quality; (c) country culture moderated the pathway from screen time to self-control (b = 0.50, p = 0.007), with the negative impact of screen time on self-control being stronger among British adolescents.

**Discussion:**

Collectively, these results suggest a potential mechanism through which entertainment screen time influences sleep quality and highlight the role of culture in this process. They underscore the importance of reducing entertainment screen time and enhancing parental control over screen time to address self-control deficits and, consequently, reduce sleep problems among adolescents.

## 1 Introduction

Sleep is ubiquitous across species and necessary for survival. Humans are estimated to spend approximately one-third of our lives sleeping to help us maintain our physical and mental health (Opp, 2009). Sleep deprivation and poor sleep quality have become a pressing concern worldwide. The 2024 Chinese National Healthy Sleep White Paper reported that Chinese residents, on average, go to bed after 12:00 am and sleep for 6.75 h per night; only 19% of respondents reported no sleep-related issues (Chinese Sleep Research Society, 2024). A cross-national survey sampled from 17 countries with a total of 36,000 respondents found that nearly four in ten individuals experience less than three nights of quality sleep per week, and 14 out of the 17 countries get less than the recommended hours of sleep at night (ResMed, 2024).

As the second peak of physiological development, adolescence is a critical period for mental health development, and a significant number of them experienced depression, anxiety and other mental health disorders (Polanczyk et al., 2015; Wang et al., 2017). A large number of studies found that adolescents need sufficient and high quality sleep for their psychological and physical well being. For example, (Oginska and Pokorski 2006) observed that sleep loss associated closely with younger adolescents' daytime sleepiness, fatigue, and negative mood. (Vermeulen et al. 2021) observed that both short sleep and problematic sleep associated closely with adolescents' worse psychological functioning. The cross-lagged models in this study also indicated bidirectional associations. Moreover, according to Jalilolghadr et al.'s (2021) study, adolescents with difficulty falling asleep and shorter sleep duration tended to have significantly lower academic performance than those with better sleep quality and longer sleep duration.

However, even though adolescence is a period in which healthy sleep is particularly important, their sleep issues, such as insufficient sleep, delayed sleep-wake behavior, and sleep disturbances, have become more widespread around the world, especially in recent 5 years. Poor sleep quality and sleep deprivation were observed in 31–65 % of adolescents no matter in western or in eastern countries (Li et al., 2020; Cavalcanti et al., 2021; Hartstein et al., 2024). After meta-analyzing 57 articles (covering 206,601 participants) carried out from 2020 to 2022, researchers found that the overall prevalence of children's and adolescents' sleep disturbances was 34.0% (Cai et al., 2024), which is 20% higher than decades ago (Morrison et al., 1992). Altogether, sleep problems in adolescents is a topic worthy of further study.

### 1.1 Screen time and sleep of adolescents

An important question concerning adolescents' sleep is whether sleep problems are increasing in line with environmental changes. Plenty of studies have observed that, in addition to biological factors, this worldwide decline in sleep duration and sleep quality among adolescents has been considerably influenced by environmental factors, particularly media use (Cain and Gradisar, 2010; Hysing et al., 2015). After a systematic review of 67 studies published between 1999 and 2014, screen time was found to be adversely associated with sleep outcomes (primarily shortened duration and delayed bedtime) in 90% of the studies (Hale and Guan, 2015). Another review, which included 49 studies published from 2009 to 2019, found that adolescents' difficulties in falling asleep and diminished sleep quality were also linked to screen time (Lund et al., 2021).

Adolescence is a time of increased independence and emergence of new social roles, which leads to much more screen time without parental control than childhood. In fact, today's children also spend a significant amount of time using digital media devices. Researchers found that the average time of television use was around 1 h or more for infants under three (Madigan et al., 2020). This increases to two and a half hours for preschoolers (Rideout and Robb, 2020; Susilowati et al., 2021), and more than 3 h for primary school students. In adolescence, the screen time increases dramatically to more than 5.5 h per day only for entertainment purposes and 20% and 41% adolescents aged 8–12 and 13–18 reported even more than 8 h entertainment screen consumption per day (Rideout, 2015). As what we can see media use has become a nearly indispensable part of children and adolescents' lives and its impact cannot be ignored.

In general, there are three hypothetical mechanisms to help understand this association. The first is that greater media exposure may displace the time that otherwise would have been used for sleep, thus shortening sleep duration and even disrupted circadian rhythm (Van den Bulck, 2004a). The second one is that blue light released from screens may arouse adolescents' alertness and suppress the hormone melatonin, thus decreasing sleepiness and deleteriously affecting sleep quantity and quality (Higuchi et al., 2003; Bowler and Bourke, 2019). Thirdly, screen content, especially violence and excitement, can be emotionally arousing, causing individuals' alertness (Van den Bulck, 2004b; Anderson and Bushman, 2002). It should be noted that existing hypothetical mechanisms primarily focus on how screen time impacts sleep through biological factors, while less attention has been given to the role psychological factors play in this relationship. The Biopsychosocial Model points out that to understand a person's health status, not only biological factors, but also psychological and social factors should be considered (Wade and Halligan, 2017). Therefore, this study will try to find out whether screen time has a negative impact on adolescents' sleep at first, if so, what role does psychological factor play in the underlying mechanism and whether there is any other moderator factor that can affect the correlation.

### 1.2 The mediating role of self-control

Self-control—defined as one's cognitive function to alter dominant responses to abide by social values and moral norms and to support the pursuit of long-term goals (Baumeister et al., 2007)—plays a key role in positive youth development (Li et al., 2019). It is an essential contributor toward success whenever adolescents face tasks that require concentration, planning, memory, problem-solving, and decision-making (Diamond, 2006). Highly self-controlled adolescents are believed to have a better relationship with teachers and classmates, engage more in classroom activities, and better control their attention and behavior as well (Jacob and Parkinson, 2015).

Less self-control was observed to increase sleep problems widely. For example, less self-control was related to more bad sleep hygiene behaviors, such as no regular sleep schedules and having alcohol, caffeine, and doing high-energy activities before bedtime (Barber et al., 2013). And the individuals with less self-control were found to sleep later than they expected or tended to finish tasks extremely late, which further increased their bedtime procrastination and delayed their bedtime as well (Nauts and Kroese, 2016).

Moreover, researchers observed that among adolescents both high (more than 7 h/day) and moderate users (4 h/day) of screens displayed significantly lower self-control than low users (1 h/day; Twenge and Campbell, 2018). According to a recent study (Miedzobrodzka et al., 2024), adolescents' TikTok use positively predicted their bedtime procrastination, more than that the participants who failed to control their screen time were more affected in bedtime procrastination [b = 0.52, 95 % CI (0.404, 0.643)] than those who were able to control their screen consumption [*b* = 0.02, 95 % CI (0.007, 0.041)]. It indicates that screen time increases adolescents' bedtime procrastination and undermines their self-control. Moreover, those who struggle to regulate their digital consumption will experience significantly greater sleep impairments compared to peers with effective self-control.

However, less is known about whether the relationship between screen time and sleep is due to the increased self-control issues caused by media use or due to less self-control triggered more screen time. Although previous research has examined relationships among media use, sleep, and self-control (e.g., Exelmans and Van den Bulck, 2018; Lin and Chung, 2022), screen consumption is rarely considered a trigger of the entire chain. According to Ecological Systems Theory, an individual's development is influenced by a series of interconnected environmental factors (Bronfenbrenner and Evans, 2000). This is an age that massive media usage is an environmental factor that cannot be underestimated.

Screen usage is argued to harm adolescents' self-control by researchers (Lissak, 2018). The reason may be due to brain plasticity. Plasticity is “the ability of an organism to react to an environmental input with a change in form, state, movement, or rate of activity” (West-Eberhard, 2003). According to brain plasticity theory, brain reshapes itself throughout life according to individual's thoughts and actions, because the repeated thoughts and actions can change the patterns of synapses and become pathways in brain (Murphy and Corbett, 2009). Therefore, we can see that if adolescents are exposed much to the real-time responses and constant connectivity provided by media use, their brain will be reformed and then they will be used to this condition and prefer to the instant gratification and lose tolerance for activities requiring sustained effort and delay of rewards, thus lose their self-control ability (George et al., 2023).

Above all, it can be inferred that self-control may play a mediating role between media use and adolescent sleep quality, but previous studies did not pay sufficient attention to this. We assume that screen time has a significant negative impact on adolescents' sleep quality (Hypothesis 1), moreover, self-control plays a mediating role in this relationship (Hypothesis 2).

### 1.3 Country as a moderating factor

We found that although the significant risk of media use for adolescents' self-control issues was observed in a considerable number of empirical investigations, the intensity of this negative influence varies across studies from different country cultures. In some studies the effect size is relatively large. For example, it has been found that high levels of screen time are strongly correlated with reduced self-control abilities among American adolescents and children. High screen users were observed to be twice as likely to frequently lose their temper compared to low screen users and 46% more likely to struggle with calming down when excited (Twenge and Campbell, 2018). However, in some studies, the effect size is relatively small. For instance, a low but statistically significant negative correlation between self-control ability and smartphone usage (*r* = −0.23, *p* < 0.001) was reported among Chinese adolescents (Xiang et al., 2020). It is noteworthy that journal articles documenting no impact of screen time on self-control among adolescents are remarkably rare. But we can still find some studies announce no significant impact of screen time on adolescents' indicator of self-control. For example, a preprint article from (Yu et al. 2025) observed no significant association between gaming time, internet gaming disorder and impulsivity among 1857 Chinese adolescents. This phenomenon may be attributed to publication bias or culture difference.

According to Bandura's Reciprocal Determinism Theory, individuals' behaviors will impact individuals, but the role of social environment in this relationship cannot be neglected (Woodcock and Tournaki, 2023). In other words, even when individuals share the same behavioral habits, the impact of these habits on each of them may not be exactly the same. This is because the different environments they live in will shape their fundamental human behaviors and social psychological processes, and cross-cultural psychologists and some social psychologists, have already provided evidence for this phenomenon (Hogg and Vaughan, 2022). It suggests that these varying social surroundings cause the same habits to have varying degrees of impact on each individual. Therefore, this study believes that the intensity of screen time's impact on adolescents may also be influenced by different culture.

Altogether, this study is interested in whether different country culture is one of the fundamental factors influencing the relationship between adolescents' screen time and self-control, and whether it further affects the relationship between screen time and sleep quality through self-control.

**Figure 1:**
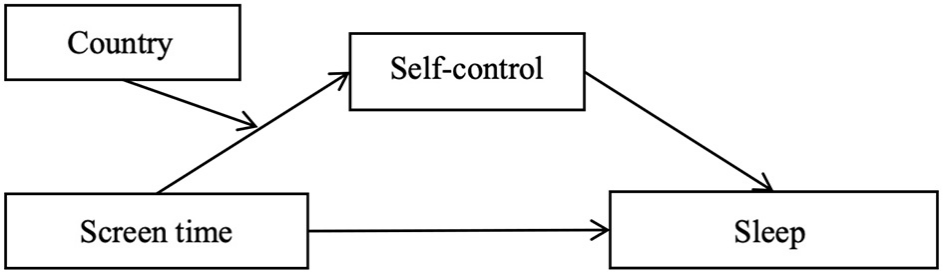
A Hypothetical model with moderated mediating effects.

More interestingly, we noticed that Western adolescents received much broader attention compared with adolescents from other parts of the world (Hale and Guan, 2015), and studies conducted with Chinese adolescents are relatively few (Li et al., 2019). Thus, in view of the significant differences between Eastern and Western cultures, this study recruited both Chinese and British adolescents to explore whether there is any difference between them. And we assume that country can moderate the relationship between screen time and self-control (Hypothesis 3) due to the different cultures. Above all, based on the relevant theories and existing literature, this study intends to reveal the impact of screen time on adolescents' sleep quality through self-control and the role of country in it. The hypothetical model is shown in Figure 1 (Hypothesis 4).

## 2 Methods

### 2.1 Participants

Convenience sampling was used to collect data of adolescent students from four schools (one primary school and one junior high school each in the UK and China), and a total of 731 students participated in the study. We did not include lower grades primary school students (1–4 grades in China, 1–5 grades in the UK), due to the age of those students are more likely to be out of the age range identified by the World Health Organization (ages 10–19; Adolescent Health, 2025) than the age of higher grades primary school students and junior high school students. All other students from the schools were sent parental consent forms to obtain permission.

As a means of meeting the purpose of the study and controlling the quality of the survey responses, the study excluded the data: (1) participants aged lower than 10 years old and higher than 19 years old; (2) pre-existing sleep or other mental health disorders; (3) missing values; (4) inconsistent survey contents. These exclusion criteria were in line with the previous studies (Wang et al., 2023a,b). Therefore, a small number of participants met these exclusion criteria, leaving 650 valid responses.

### 2.2 Procedures

To ensure participant comparability, this study selected Tianjin, China, and Leeds, UK, for participant recruitment as they share comparable levels of socioeconomic development. Firstly, investigators contacted all accessible public primary and junior high schools within the urban areas of these two cities via email. Then visited the schools interested in the present study, explained the purpose of the study to teachers, and sent the protocol. At last, 4 schools were finally selected. Prior to the commencement of the study the parental consent was sent and gathered by the schools. The detailed information regarding the research objectives, procedures, and privacy protection measures were provided. Only the students both themselves and their guardians agreed to participate, could complete the research.

**Figure 2:**
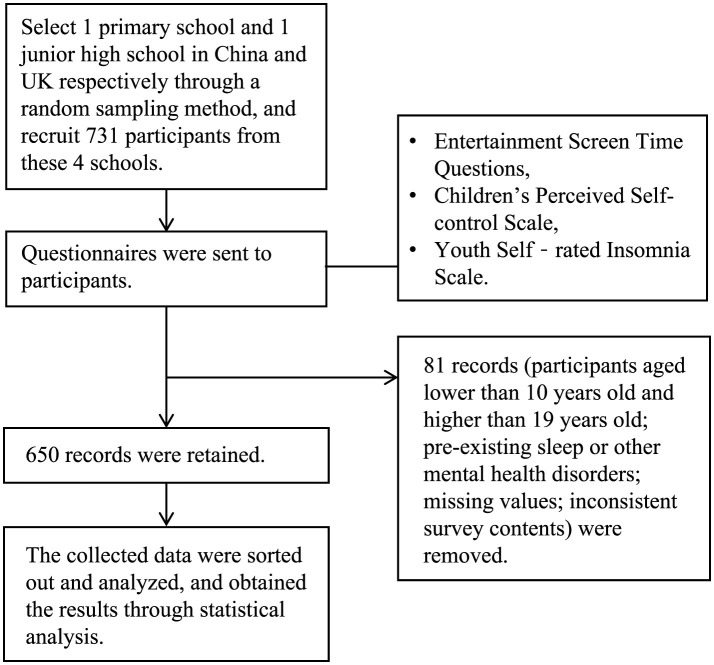
Study flowchart.

All of the research were conducted in computer classrooms using computers monitored and supported by teachers who had been trained by the researchers. Before the survey took place, instructions were read aloud by teachers to remind students that withdrawal was permitted during the study process. Responses to the survey were kept confidential, and the present study was approved by university. The flowchart is shown in Figure 2. This study was approved by the scientific research ethics committee of university.

### 2.3 Measurements

#### 2.3.1 Screen time for entertainment purposes

Screen time for entertainment purposes was measured by using a five-item scale. These items assessed the time adolescents spent on playing video games, watching television, using computer, smartphone, and tablet for entertainment purposes on each typical school day (1 = never, 2 = less than half an hour, 3 = 0.5–1 h, 4 = 1–2 h, 5 = 2–3 h, 6 = 3–4 h, 7 = 4–5 h, and 8 = more than 5 h). Mean screen time was calculated. We added together the scores of each screen time and then divided the score by 5, with a higher score indicating a more screen time.

#### 2.3.2 Self-control

Self-control problems were assessed by using a 11-item Children's Perceived Self-control Scale with dichotomous “yes/no” answers (Humphrey, 1982). Nine items were rated 0 (Yes) or 1 (No). Two items were rated inverted. The total score is calculated by adding up the scores of all items, and the higher the score, the higher the degree of self-control ability. The scale demonstrated satisfactory reliability and validity in a number of prior studies (Chen et al., 2023; Huang et al., 2018).

#### 2.3.3 Sleep quality

To assess adolescents' sleep quality, we selected 3 items from Youth Self-Rating Insomnia Scale (YSIS). YSIS was developed to assess insomnia severity in the past month in adolescents. It has shown satisfactory reliability and validity in a number of studies in adolescents (Liu et al., 2019; Hu et al., 2024). The items selected are “During the past month, how often would you say you have had trouble falling asleep?” “During the past month, how often would you say you haven't have enough sleep?” “During the past month, how often would you say you have felt unrested and unrestored upon waking?”. Each item is rated on a 5-point scale ranging from 1 (very often) to 5 (never). The scale score was computed by summing the item scores and then divided by 3, with a higher score indicating a higher level of sleep quality. The Cronbach's alpha coefficient in this study was 0.74, indicating good reliability.

### 2.4 Data analysis

All analyses in this study were conducted using SPSS 22.0 and Hayes's PROCESS version 3.0.

Normality was satisfied. First, descriptive statistics were calculated for sociodemographic information, while Pearson correlations were adopted to examine the relationships among screen time, self-control, and sleep quality. Second independent samples t-tests were used to test for the difference between Chinese and British adolescents. Then, a hierarchical multiple linear regression was carried out to evaluate the predictive impact of screen time on self-control and to examine the moderating role of country in the correlation between them. Last, the PROCESS macro in SPSS was used to test for the mediation and moderating effects, with 5,000 bootstrap samples. Significant parameters were identified as those with 95% confidence intervals (CI) for regression coefficients that did not include 0. The procedure involves the following steps: (a) Specify the independent variable (screen time), dependent variable (sleep quality), and the mediator variable (self-control); (b) Use the PROCESS macro to define the model number (Model 4) that supports one mediation variable; (c) Set the number of bootstrap samples as 5,000; (d) All variables were mean-centered prior to the analyses. (e) Run the analysis to obtain the path coefficients and significance levels for the mediation step. (f) Then, we utilized Model 7 in PROCESS to test the moderated mediation model, with “country” as a moderator. The categorical variable “country” was converted into dummy variables, with 0 assigned to the UK and 1 to China.

## 3 Results

### 3.1 Descriptive statistics of sample characteristics

A total of 650 participants were included in the analysis of the present study. The average age of the sample was 12.93 years, with a standard deviation of 0.96, ranging from 10–16 years. The total sample comprised 48.2% male students (*n* = 313) and 84.2% students from China (*n* = 541). The mean age (*SD*) of Chinese participants was 13.01 (0.98) years, ranging from 10 to 18, with 48.2% male students (*n* = 261); The mean age (*SD*) of British participants was 12.53 (0.63) years, ranging from 12 to 14, with 47.7% male students (*n* = 52).

### 3.2 Correlation analyses

Table 1 displays the means, standard deviations, and Pearson correlation coefficients for the variables under investigation. The research results indicate that there is a significant negative correlation between adolescents' screen time and their self-control (*r* = −0.27, *p* < 0.001), as well as between screen time and sleep quality (*r* = −0.30, *p* < 0.001). On the other hand, self-control and sleep quality are positively correlated with each other (*r* = 0.21, *p* < 0.001). It indicates that the more time adolescents spent on screens for entertainment purposes the more issues they will get for their self-control and sleep quality, and the adolescents who have more self-control problems may also have more sleep issues.

**Table 1:** Descriptive statistics and Pearson correlations of the studied variables.

**Variables**	***Mean***	***SD***	**1**	**2**	**3**
1. Screen time	2.94	1.32	1		
2. Self-control	5.50	2.09	−0.27^***^	1	
3. Sleep quality	3.27	0.97	−0.30^***^	0.21^***^	1

*n* = 650, ^*^p < 0.05, ^**^p < 0.01, ^***^p < 0.001 (Two-Tailed Test).

### 3.3 Independent-samples *t* tests

We conducted independent-samples *t* tests between Chinese and British adolescents to examine the between-group differences in their screen time, self-control and sleep quality (see Table 2). Results revealed a significant difference in screen time between countries (*t* = −4.47, *p* < 0.001). British adolescents (*Mean* = 3.45, *SD* = 1.10) reported significantly more screen time for entertainment purposes than Chinese adolescents (*Mean* = 2.84, *SD* = 1.33). No significant difference was observed in self-control (*p* = 0.060) and sleep quality (*p* = 0.180), which indicates that self-control and sleep quality between Chinese and British adolescents are not significantly different.

**Table 2:** Descriptive statistics and independent samples t-tests between Chinese and British adolescents.

**Variables**	**Screen time**		**Self-control**		**Sleep quality**	
	***Mean*** ±***SD***	***t***	***Mean*** ±***SD***	***t***	***Mean*** ±***SD***	***t***
China	2.84 ± 1.33	−4.47^***^	5.57 ± 2.36	1.92	3.29 ± 0.99	1.35
The UK	3.45 ± 1.10		5.15 ± 2.36		3.16 ± 0.87	

*n* = 650, ^*^p < 0.05, ^**^p < 0.01, ^***^p < 0.001.

### 3.4 Mediation analyses

As shown in Table 3, the results of hierarchical multiple line regression analysis are presented. In Model 4 of Table 3, screen time has a significant negative impact on sleep quality (*b* = −0.22, *p* < 0.001), thus confirming the validity of Hypothesis 1. Moreover, as shown in Model 1 and Model 5 of Table 3, screen time was negatively associated with self-control (*b* = −0.43, *p* < 0.001), and self-control positively predicted sleep quality (*b* = 0.07, *p* < 0.001).

**Table 3:** Hierarchical multiple line regression results.

**Variables**	**Self-control as a dependent variable**			**Sleep quality as a dependent variable**	
	**Model 1**	**Model 2**	**Model 3**	**Model 4**	**Model 5**
	***b***	***b***	***b***	***b***	***b***
Screen time	−0.43^***^	−0.43^***^	−0.87^***^	−0.22^***^	−0.19^***^
Country		0.13	−1.58		
Screen^*^Country			0.50^**^		
Self-control					0.07^***^
R^2^	0.07	0.08	0.09	0.09	0.11
F	51.57	25.94	19.88	61.92	38.41

*n* = 650, ^*^p < 0.05, ^**^p < 0.01, ^***^p < 0.001 (Two-Tailed Test).

To further exam the mediating role of self-control, Model 4 in the PROCESS macro by Hayes was used to examine the mediation effect of self-control on the relationship between screen time and sleep quality. As shown in Table 4, the 95% confidence interval for the mediating effect of self-control does not include zero [95% CI = (−0.05, −0.01)]. This indicates a significant mediating effect (*b* = −0.03), thus supporting Hypothesis 2. Additionally, after controlling for self-control ability, the direct effect of screen time on sleep was −0.19 [95% CI = (−0.25, −0.13)], which remains significant. This demonstrates that self-control partially mediates this relationship.

**Table 4:** Index of the mediation model.

**Sleep quality as a dependent variable**	***b* [95% CI]**	**Boot*SE***
Total effect	−0.22 [−0.28, −0.17]	0.03
Direct effect	−0.19 [−0.25, −0.13]	0.03
Indirect effect	−0.03 [−0.05, −0.01]	0.01

Independent variable is screen time; Mediation variable is self-control.

### 3.5 Moderated mediation analyses

**Figure 3:**
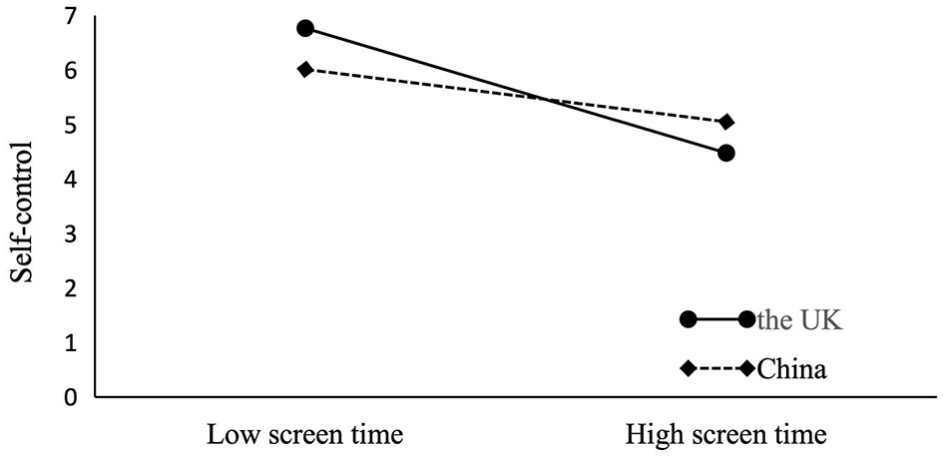
The interaction effect of screen time and country on self-control.

As shown in Model 3 of Table 3, the interaction between screen time and country has a significant positive effect on self-control ability (*b* = 0.50, *p* = 0.007). This means that country serves as a significant positive moderator in the relationship between screen time and self-control. Therefore, Hypothesis 3 is supported. A simple slope test was performed to reveal how different country culture moderates the impact of screen time on self-control. The categorical variable “country” was converted into dummy variables, with 0 standing for the UK and 1 for China. According to Figure 3, screen time has a significant negative predictive effect on self-control ability in Chinese adolescents [*b* = −0.37, *p* < 0.001, 95% CI = (−0.49, −0.24)]; Yet, for British adolescents, screen time has a significant negative but stronger predictive effect on self-control [*b* = −0. 87, *p* < 0. 001, 95% CI = (−1.22, −0.53)].

To test Hypothesis 4, Model 7 in the PROCESS macro was used to examine the role of country in the relationship between screen time and sleep quality, with self-control acting as a mediator (see Table 5). The results revealed that the indirect effect of screen time on sleep quality through self-control is significantly negative in Chinese adolescents [*b* = −0.24, *SE* = 0.01, 95% CI: (−0.04, −0.01)]. While, in British adolescents, this relationship is stronger [*b* = −0.58, *SE* = 0.02, 95% CI: (−0.11, −0.02)]. It suggests that country moderates the indirect relationship between screen time and sleep quality via self-control. The negative impact from screen time on adolescents' sleep quality is more stronger in British adolescents than in Chinese adolescents. Hypothesis 4 is supported.

**Table 5:** Index of the moderated mediation model.

**Sleep quality as a dependent variable**	***b* [95% CI]**	**Boot*SE***
The UK	−0.58 [−0.11, −0.02]	0.02
China	−0.24 [−0.04, −0.01]	0.01

Independent variable is screen time; Mediation variable is self-control.

## 4 Discussion

Through the examination of the moderated mediation model, this study explores the pathway through which adolescents' entertainment screen time impacts their sleep quality by influencing self-control and identifies the conditions under which the interaction among screen time, self-control, and sleep quality is most pronounced. The principal findings are as follows: (1) a positive correlation between screen time and sleep quality was observed; (2) self-control served as a mediator in the relationship between screen time and sleep quality; and (3) country culture played a moderating role in the connection between screen time and self-control, indicating that British adolescents experience a relatively higher level of self-control problems resulting from heavy screen consumption.

### 4.1 The influence of screen time for entertainment purposes on adolescents' sleep quality

Consistent with prior investigations (Hysing et al., 2015; Hale and Guan, 2015; Lund et al., 2021), our study establishes a negative association between screen time and sleep quality, supporting the notion that heavy screen consumption for entertainment purposes is a risk for adolescents' sleep problems. In fact, over the past decades, excessive screen time has been found to have negative impacts on not only the sleep quality but also the sleep quantity and sleep outcomes of adolescents worldwide, such as short sleep duration, delayed bedtime, prolonged sleep onset latency, insomnia, and daytime tiredness. Moreover, this relationship is not limited to adolescents but is also observed among children (Lund et al., 2021) and adults (Murphy and Corbett, 2009). Hence, implementing restrictions targeting media use, especially limiting screen time and controlling media content, is crucial for addressing sleep issues in adolescents and, more broadly, in humanity as a whole.

### 4.2 The mediating role of self-control

Complementing current research try to explain the underlying mechanism in the relationship between screen time and sleep quality from biological factors (Van den Bulck, 2004a; Bowler and Bourke, 2019), our study unveils a novel psychological mediator, elucidating how entertainment screen time might decrease self-control, consequently leading to sleep problems. Drawing insights from established literature (Lissak, 2018) and the brain plasticity theory (Murphy and Corbett, 2009), repeated actions will change the patterns of individuals' synapses and become pathways in brains, further change humans' activities and functions. Therefore, much entertainment screen consumption will lead adolescents to be used to the fast-paced, exciting, interesting contents and the real-time responses provided by media use, resulting in attenuated self-control in activities requiring sustained effort and delay of rewards. Moreover, this study demonstrated that self-control lose will increase sleep problems further, which is consistent with previous research. As what those research announced, this correlation may be due to adolescents' bedtime procrastination, no regular sleep schedules or other bad sleep hygiene behaviors related to low self-control (Barber et al., 2013; Nauts and Kroese, 2016). Taken together, our findings underscore the potential for entertainment screen time to diminish self-control and consequently lead to sleep problems in adolescents.

### 4.3 The moderating role of country culture

Our study identified country culture as a significant moderator in the relationship between screen time and self-control. Specifically, compared to Chinese adolescents, excessive screen use has a greater negative impact on self-control in British adolescents, which in turn negatively affects their sleep quality, thereby validating Hypothesis 3 and Hypothesis 4. This finding is in line with some prior research, which found a relatively lower influence from screen time on self-control in Asian country, and a relatively higher influence in Western country. For example, in Xiang and colleagues' study, there was a low negative correlation between Chinese adolescents' self-control and smartphone usage (*r* = −0.23, *p* < 0.001) (Xiang et al., 2020), but in Twenge and Campbell's study US high users of screens were observed twice as likely to often lose their temper than low users of screen and 46% more likely to not be able to calm down when excited (Twenge and Campbell, 2018).

One potential explanation for this country disparity lies in cultural difference, which will further impacts parent control on adolescents' entertainment screen time. It's widely known that Chinese culture is rooted in collectivism, whereas British culture emphasizes individualism and personal goals. Chinese parents tend to place higher expectations on their children's academic achievements compared to British parents (Zhao et al., 2015). This cultural difference will result in varying levels of parental control over children's after-school activities. Researchers observed that Chinese parents are more likely to guide children's after school activities, including limit their screen time (Newman et al., 2007). In contrast, British parents are more likely to let their children choose after-school activities autonomously (Newman et al., 2007).

Although this study did not exam if Chinese adolescents have more parents control on their screen time than British adolescents, a significant difference in screen time between British and Chinese adolescent was observed. According to the results, British adolescents do spend significantly more time on screens for entertainment purposes than Chinese adolescents (*t* = −4.47, *p* < 0.001). This is consistent with another cross-cultural study conducted in 12 countries, including Mainland China and the UK, found that Chinese children (1.9 h per day) spent significantly less time on television, video games, and computers than British children (2.9 h per day) (LeBlanc et al., 2015). Consequently, cultural differences may play a key role in parental control on adolescents' screen time for entertainment purposes, which will further influence self-control.

### 4.4 Limitations and future recommendations

Several limitations of this study should be acknowledged. First, although this study aimed to balance the number of British and Chinese participants by selecting one primary school and one junior high school in each country and sending consent forms to all students who met the criteria from the four selected schools, we received a significant small number of positive feedbacks from British students than from Chinese students. Thus, the number of participants from each country was not balanced. Future cross-cultural research should take geographical differences into account and try to balance the number of participants from different cultures. Second, the cross-sectional nature of the research design does not allow causal inferences. It should be noted that although screen time is modeled as an independent predictor of self-control in the present study, there are still some studies obversed less self-control predicted greater screen consumption, suggesting a potential bidirectional relationship between them. Future investigations would benefit from employing longitudinal or experimental designs to more effectively elucidate the direction of causality. Third, the study relied on self-report measures, which might be subject to biases such as social desirability. Future research could be enhanced by integrating objective data, such as sleep diary, and other-rated data, including evaluations from parents, peers, and teachers, to achieve a more comprehensive and objective analysis.

### 4.5 Research implications

Despite these limitations, the current findings enhance our understanding of the relationship between adolescents' sleep problems and their entertainment screen time, providing valuable insights for future adolescent media usage strategies. We identified self-control as a mediating factor and country culture as a moderating influence, delineating how and under what conditions screen time relates to self-control and further influences sleep problems. Importantly, the role of country culture in the impact of screen time on self-control was emphasized. Thus, our findings carry significant implications for developing restrictions to address sleep issues in adolescents. Given the mediating role of self-control, restrictions could integrate strategies, such as goal-setting and self-monitoring, to reduce the negative influence to sleep quality caused by excessive entertainment screen time. Furthermore, the moderating influence of national culture highlights how Chinese culture can reduce the negative effects of screen time on self-control. As discussed above, Chinese parents' monitoring of their children's screen time for entertainment purposes should be a key factor. We therefore propose more attention on adolescents' entertainment screen time and more parents' control on it.

## 5 Conclusion

In summary, our study proposed a moderated mediation model to explore how screen time may influence sleep quality in Chinese and British adolescents, suggesting potential roles of self-control and culture differences. Our findings indicate that heavy screen consumption for entertainment could pose a risk for adolescents' sleep quality through reducing self-control. Moreover, country culture may moderate the extent to which screen time influences self-control. Based on these findings, this study offers insights into limiting adolescents' media usage time, particularly through parental control, aimed at addressing their sleep issues and improving sleep quality.

## Data Availability

The raw data supporting the conclusions of this article will be made available by the authors, without undue reservation.
